# Rapid detection of *Pseudomonas aeruginosa* by recombinase polymerase amplification combined with CRISPR-Cas12a biosensing system

**DOI:** 10.3389/fcimb.2023.1239269

**Published:** 2023-08-10

**Authors:** Shuang Liu, Siyuan Huang, Fang Li, Yuanyuan Sun, Jin Fu, Fei Xiao, Nan Jia, Xiaolan Huang, Chunrong Sun, Juan Zhou, Yi Wang, Dong Qu

**Affiliations:** ^1^ Department of Critical Medicine, Children’s Hospital Affiliated Capital Institute of Pediatrics, Beijing, China; ^2^ Experimental Research Center, Capital Institute of Pediatrics, Beijing, China

**Keywords:** *Pseudomonas aeruginosa*, recombinase polymerase amplification, RPA, CRISPR-Cas12a, *oprL*

## Abstract

*Pseudomonas aeruginosa* (*P. aeruginosa*) is an important bacterial pathogen involved in a wide range of infections and antimicrobial resistance. Rapid and reliable diagnostic methods are of vital important for early identification, treatment, and stop of *P. aeruginosa* infections. In this study, we developed a simple, rapid, sensitive, and specific detection platform for *P. aeruginosa* infection diagnosis. The method integrated recombinase polymerase amplification (RPA) technique with clustered regularly interspaced short palindromic repeat (CRISPR)–CRISPR-associated protein 12a (Cas12a) biosensing system and was termed *P. aeruginosa*–CRISPR–RPA assay. The *P. aeruginosa*–CRISPR–RPA assay was subject to optimization of reaction conditions and evaluation of sensitivity, specificity, and clinical feasibility with the serial dilutions of *P. aeruginosa* genomic DNA, the non*–P. aeruginosa* strains, and the clinical samples. As a result, the *P. aeruginosa*–CRISPR–RPA assay was able to complete *P. aeruginosa* detection within half an hour, including RPA reaction at 42°C for 20 min and CRISPR-Cas12a detection at 37°C for 10 min. The diagnostic method exhibited high sensitivity (60 fg per reaction, ~8 copies) and specificity (100%). The results of the clinical samples by *P. aeruginosa*–CRISPR–RPA assay were consistent to that of the initial result by microfluidic chip method. These data demonstrated that the newly developed *P. aeruginosa*–CRISPR–RPA assay was reliable for *P. aeruginosa* detection. In summary, the *P. aeruginosa*–CRISPR–RPA assay is a promising tool to early and rapid diagnose *P. aeruginosa* infection and stop its wide spread especially in the hospital settings.

## Introduction

*Pseudomonas aeruginosa* (*P. aeruginosa*) is a Gram-negative, rod-shaped aerobe/facultative anaerobe belonging to the genus *Pseudomonas* of family *Pseudomonadaceae*. Metabolically, *P. aeruginosa* is versatile and can adapt to a wide range of niches, including soil, aquatic environment, plants, animals, and human beings (Silby et al., 2011; Gellatly and Hancock, 2013; Moradali et al., 2017). *P. aeruginosa* is an important opportunistic pathogen of human beings, especially for the vulnerable patients with cystic fibrosis (CF) lungs (Turner et al., 2015), obstructive pulmonary diseases (Eklöf et al., 2020), and other immunocompromised and hospitalized patients (Malhotra et al., 2019). It infects three-quarters of patients with CF and leads to high morbidity and mortality rate (Surette, 2014). Meanwhile, *P. aeruginosa* is the predominant pathogen causing otitis media (Mittal et al., 2015), keratitis (Hilliam et al., 2020), endocarditis (Sheppard, 1991), bacteremia (Fabre et al., 2019), burn and wound infections (Salerian, 2020), urinary tract infections (Yin et al., 2022), and more. However, treatment of *P. aeruginosa* infection in clinical setting has confronted with great challenge due to its resistance to different antibiotics and antiseptic (Lister et al., 2009; Chevalier et al., 2017). A combination of intrinsic, acquired, and adaptive ability of *P. aeruginosa* to counter antibiotic attack (Pang et al., 2019) and its extensive reservoirs in nosocomial and community environments (Ratnam et al., 1986; van Asperen et al., 1995; Yakupogullari et al., 2008; Quick et al., 2014) complicated the effective treatment and control of *P. aeruginosa* infection and rendered it a healthcare concern (Rosenthal et al., 2016). *P. aeruginosa* has become one of the notorious “ESKAPE” pathogens (Pendleton et al., 2013) and been considered as the “critical” category of the World Health Organization’s priority list of bacterial pathogens for which research and development of new antibiotics is urgently needed (Tacconelli et al., 2018). Under this context, development of rapid, accurate, and sensitive detection method for *P. aeruginosa* is of vital importance and urgently required for early diagnosis of *P. aeruginosa* infection and effective control its wide spread.

In clinical settings and routine laboratories, specimen culture is the most common and gold standard for *P. aeruginosa* identification (Rytter et al., 2020). Use of culture-based method is able to determine antibiotic susceptibility; however, obtaining the results usually takes a minimum time of 48 h, which may delay antibiotic treatment and compromise patient outcome (Rytter et al., 2020). In this regard, more rapid and sensitive diagnostic tests for *P. aeruginosa* detection are still urgently needed. During the past decades, a plenty of polymerase chain reaction (PCR)–based techniques, including conventional PCR and real-time PCR methods, have been widely developed and applied in pathogen identification including *P. aeruginosa* (Williams et al., 2010; Lim et al., 2021). Although sensitive and specific, these methods usually rely on sophisticated instruments and well-trained technicians, which commonly equipped in the well-established laboratories and normally cost more than 1 h to report results. More recently, several isothermal nucleic acid amplification techniques that overcome the limitations of PCR-based methods have been reported for *P. aeruginosa* detection, such as recombinase polymerase amplification (RPA) assay (Yang et al., 2021), multiple–cross-displacement amplification (MCDA) assay (Li et al., 2020; Wang et al., 2020), and loop-mediated isothermal amplification assay (Takano et al., 2019). These tools could rapidly, accurately, sensitively, and specifically identify and characterize *P. aeruginosa* only with a simple an apparatus that could maintain a constant temperature (Wang et al., 2015), which demonstrated the potential to be applied in resource-limited or rural regions. However, the results identification and reporting systems of the isothermal amplification techniques normally rely on indicator, fluorescent dye or fluorescent probe, and amplification bias and non-specific amplification inherent in exponential strategies (Zhao et al., 2015). Therefore, the urgent need for new nucleic acid detection techniques with rapidity, accuracy, and high sensitivity still exists.

Discovery of the clustered regularly interspaced short palindromic repeats (CRISPR) and CRISPR-associated protein (Cas) (CRISPR-Cas) system has revolutionized the biosensing field and sparked great interest in nucleic acid detection technologies (Li et al., 2019). The CRISPR-Cas biosensing system could transfer the sequence information of targets to detectable signals (such as fluorescence or colorimetric values) by employing the collateral cleavage activities of the Cas effectors (Cas12a, Cas12b, Cas13, and Cas14), conferring this technology high sensitivity and specificity of detection and simplicity to develop, which also exhibits great potential in point-of-care tests (Gootenberg et al., 2018; Myhrvold et al., 2018; Bonini et al., 2021; Jirawannaporn et al., 2022; Kumaran et al., 2023; Zheng et al., 2023). In particular, by coupling isothermal amplification procedure, the detection performance of CRISPR-Cas biosensing system is greatly improved, and the target type also can be converted (Li et al., 2019; Zhang et al., 2023). Recently, the CRISPR-Cas–based biosensing detection platforms, such as SHERLOCK (Specific High Sensitivity Enzymatic Reporter Unlocking, RPA combination with Cas13a) (Myhrvold et al., 2018) and DETECTR (DNA Endonuclear Targeted Crispr Trans Reporter, RPA combination with Cas12a) (Chen et al., 2018), have been rapidly developed and already commercial available for pathogen detection.

In this study, a CRISPR-Cas12a–based RPA detection platform (CRISPR-RPA) targeting the *oprL* gene was developed and validated for rapid, accurate, sensitive, and specific diagnosis of *P. aeruginosa* infection. This two-step detection platform included *oprL* gene amplification using RPA assay at 42°C within 20 min and CRISPR-Cas12a detection at 37°C for 10 min. The result was interpreted using real-time fluorescence analysis using the single-strand DNA (ssDNA) reporter (5′-FAM-TTATTAT-BHQ1-3′). The detection performance of the *P. aeruginosa*–CRISPR–RPA assay was confirmed with DNA templates of *P. aeruginosa* strains, other respiratory pathogen strains, and clinical samples.

## Materials and methods

### Reagents and apparatus

Recombinase polymerase–based amplification kit for isothermal amplification was purchased from Msunflowers Biotech Co., Ltd. (Beijing, China). The 100-bp DNA marker and the EasyPure^®^ Genomic DNA Kit for genomic DNA extraction and purification were obtained from TransGene Biotech Co., Ltd. (Beijing, China). EnGen^®^ Lba Cas12a (Cpf1) and NEBuffer r2.1 were purchased from New England Biolabs (Beijing, China). The ABI 7500 FAST real-time PCR platform (Applied Biosystems, USA) was used as the fluorescence reader. An imaging system (Gel Doc XR C, Bio-Rad, USA) was used for gel image taken.

### Bacterial strains and clinical samples

A total of 25 strains, including eight *P. aeruginosa* strains and 17 non*–P. aeruginosa* strains, were used in this study (**Table 1**). Genomic DNA of all the strains was extracted and purified using the EasyPure^®^ Genomic DNA Kit according to the manufacturer’s instruction, and species identification was confirmed by PCR amplification of the 16S rRNA gene with primer pair 27F/1492R (Neilan et al., 1997). In addition, 96 bronchoalveolar lavage fluid (BALF) samples suspected of respiratory infection were included in this study as well. DNA templates of these BALF samples were obtained by using the nucleic acid extraction reagent of Capital BioTech Co., Ltd. (Sichuan, China). All the DNA templates were stored at −20°C before use.

**Table 1:** Bacterial strains used in this study.

Bacteria	Strain no. (source of strain)^a^	No. of strains
*Pseudomonas aeruginosa*	Isolated strains (CDC)	8
*Enterococcus faecium*	Isolated strains (CDC)	1
*Shigella sonnei*	Isolated strains (CDC)	1
*Citrobacter freundii*	Isolated strains (CDC)	2
*Moraxella catarrhalis*	Isolated strains (CDC)	1
*Escherichia coli*	Isolated strains (CDC)	1
*Salmonella enteritidis*	Isolated strains (CDC)	2
*Bacillus cereus*	Isolated strains (CDC)	1
*Klebsiella pneumoniae*	Isolated strains (CDC)	1
*Streptococcosis suis*	Isolated strains (CDC)	2
*Stenotrophomonas maltophilia*	Isolated strains (CDC)	1
*Corynebacterium striatum*	Isolated strains (CDC)	1
*Streptococcus salivarius*	Isolated strains (CDC)	1
*Streptococcus pyogenes*	Isolated strains (CDC)	1
*Nocardia asteroids*	Isolated strains (CDC)	1

a CDC, Chinese center for disease control and prevention.

### Primer and crRNA design

**Figure 1:**
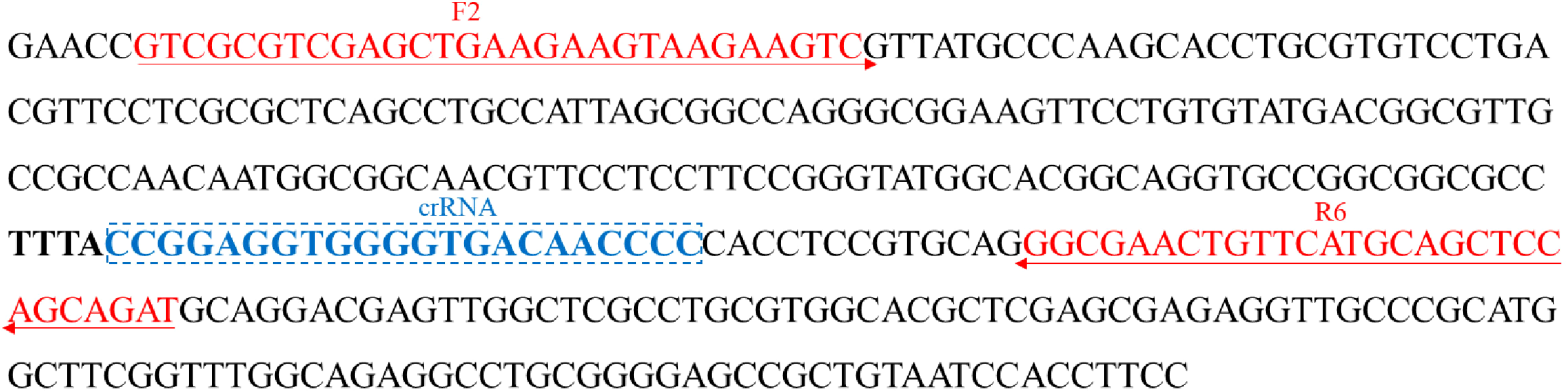
Sequences and locations of the oprL gene of *P. aeruginosa* used to design the RPA primers and crRNA. Locations of RPA primers are underlined, and crRNA is in the box. The right arrow and left arrow represent the sense and complementary sequence used in this study, respectively.

Primers for *P. aeruginosa* detection targeting *oprL* gene (GenBank: Z50191.1) (De Vos et al., 1997; Wang et al., 2020) were designed by using the Primer-Blast tool of National Center for Biotechnology Information (NCBI) on the basis of RPA reaction mechanism (Piepenburg et al., 2006). Two forward primers (F1 and F2) and six reverse primers (R1 to R6) were obtained, resulting to six pairs of primers (F1R1, F1R2, F2R3, F2R4, F2R5, and F2R6). Each primer pair was subjected to specificity assessment using the BLAST tool. After primers screen, the CRISPR RNA (crRNA) and probe were designed according to the principle of CRISPR-Cas12a effector. The probe was an ssDNA reporter that labeled with 5-Carboxyfluorescein (FAM) fluorophore and Black Hole Quencher 1 (BHQ1) quencher at the 5′ and 3′ end, respectively. Sequences and locations of all the oligonucleotides and crRNA were shown in **Table 2** and **Figure 1**, and all of them were synthesized by TianyiHuiyuan Biotech Co., Ltd. (Beijing, China).

**Table 2:** Primers, crRNA, and probe design in this study.

Primers	Sequences (5′-3′)	Length
F1	AACAATGGCGGCAACGTTCCTCCTTCCGG	29 nt
F2	GTCGCGTCGAGCTGAAGAAGTAAGAAGTC	29 nt
R1	ATCTGCTGGAGCTGCATGAACAGTTCGCC	29 nt
R2	AGCCAACTCGTCCTGCATCTGCTGGAGCT	29 nt
R3	CAACGCCGTCATACACAGGAACTTCCGCC	29 nt
R4	TGTTGGCGGCAACGCCGTCATACACAGGA	29 nt
R5	GGAAGGAGGAACGTTGCCGCCATTGTTGG	29 nt
R6	ATCTGCTGGAGCTGCATGAACAGTTCGCC	29 nt
crRNA	UAAUUUCUACUAAGUGUAGAUCCGGAGGUGGGGUGACAACCCC	43 mer
Probe	FAM-TATTATTATTATTATTT-BHQ1	17 mer

### Standard RPA amplification

According to the manufacturer’s instruction, amplification of the *oprL* gene was performed in a 50-µL reaction mixture at 39°C for 40 min. In brief, 29.5 µL of A buffer and 2 µL of each of forward and reverse primer (10 µM) were added into a tube containing lyophilized RPA enzyme mix until fully dissolved, and, then, 2 µL of template and 2.5 µL of magnesium acetate (B buffer) were added before incubated at 39°C for 40 min. The RPA products were examined by using electrophoresis on 2% agarose gel, and the images were taken by using an imaging system.

### CRISPR-Cas12a detection

The CRISPR-Cas12a detection procedure included two steps: formation of CRISPR-Cas12a–crRNA binary complex and CRISPR-Cas12a trans-cleavage reaction. The CRISPR-Cas12a–crRNA binary complex was prepared by incubating 100 nM CRISPR-Cas12a and 100 nM crRNA in 2× NEBuffer r2.1 at 37°C for 10 min and then immediately used or stored at 4°C for no more than 24 h. The CRISPR-Cas12a trans-cleavage reaction was carried out at 37°C for 10 min in a 100-µL mixture, including 18 µL of CRISPR-Cas12a–crRNA binary complex, 50 µL of 2× NEBuffer r2.1, 2.5 µL of probe (10µM), 2 µL of RPA products, and 27.5 µL of distilled water (DW). The result was monitored in a real-time manner by the real-time fluorescence detector.

### Optimization of *P. aeruginosa*–CRISPR–RPA assay

To determine the optimal reaction temperature at RPA reaction stage, RPA products amplified at temperatures ranging from 37°C to 42°C (interval of 1°C) were tested. Furthermore, the optimal reaction time for RPA reaction was detected by performing RPA reaction at optimal temperature for 10 to 40 min (interval of 10 min), respectively. The optimal RPA reaction conditions were decided according to the brightness and sharpness of target band on gel electrophoresis images.

To optimize the performance of CRISPR-Cas12a trans-cleavage reaction, a series of reaction conditions were examined as well, including the reaction volume (50 µL versus 100 µL), the trans-cleavage temperature (37°C to 42°C, with an interval of 1°C) and time (10 to 20 min, with an interval of 5 min). The optimum conditions were determined according to the fluorescence intensity with different volume, at different temperature or within different time.

In addition, to verify the true component that works in the CRISPR-Cas12a trans-cleavage reaction mixture, reactions with different combinations of CRISPR-Cas12a, crRNA, probe, and RPA products were carried out. Result was recorded by the real-time fluorescence detector.

### Sensitivity and specificity of the *P. aeruginosa*–CRISPR–RPA assay

To determine the sensitivity of the CRISPR-RPA assay for *P. aeruginosa* detection, genomic DNA of *P. aeruginosa* strains was 10-fold serially diluted from 6 ng to 0.6 fg as templates, with negative control and blank control detected simultaneously. Moreover, a total of 17 non–*P. aeruginosa* strains (**Table 1**) were employed in this study for specificity evaluation. Each test was repeated three times to ensure stability.

### Clinical validity of the *P. aeruginosa*–CRISPR–RPA assay

The *P. aeruginosa*–CRISPR–RPA assay was performed with a total of 96 BALF samples to evaluate its feasibility in clinical settings. The BALF samples were collected from patients suspected of respiratory infection in the Capital Institute of Pediatrics and had been examined for pathogen identification using microfluidic chip (MFC) technology. Of the 96 BALF samples, 19 were detected as *P. aeruginosa*–positive with MFC method. The performance of the *P. aeruginosa*–CRISPR–RPA assay was compared with that of the MFC method for *P. aeruginosa* detection.

## Results

### Confirmation of the *P. aeruginosa*–CRISPR–RPA assay for *P. aeruginosa* detection

A total of six pairs of primers were designed and employed to amplify partial sequence of the *oprL* gene. According to the results of gel electrophoresis image (**Figure S1**), primer pair F2/R6 resulted in bright and single band and exhibited excellent amplification effect. Thus, primers F2 and R6 was used for the following RPA reaction with a length of 250 bp.

**Figure 2:**
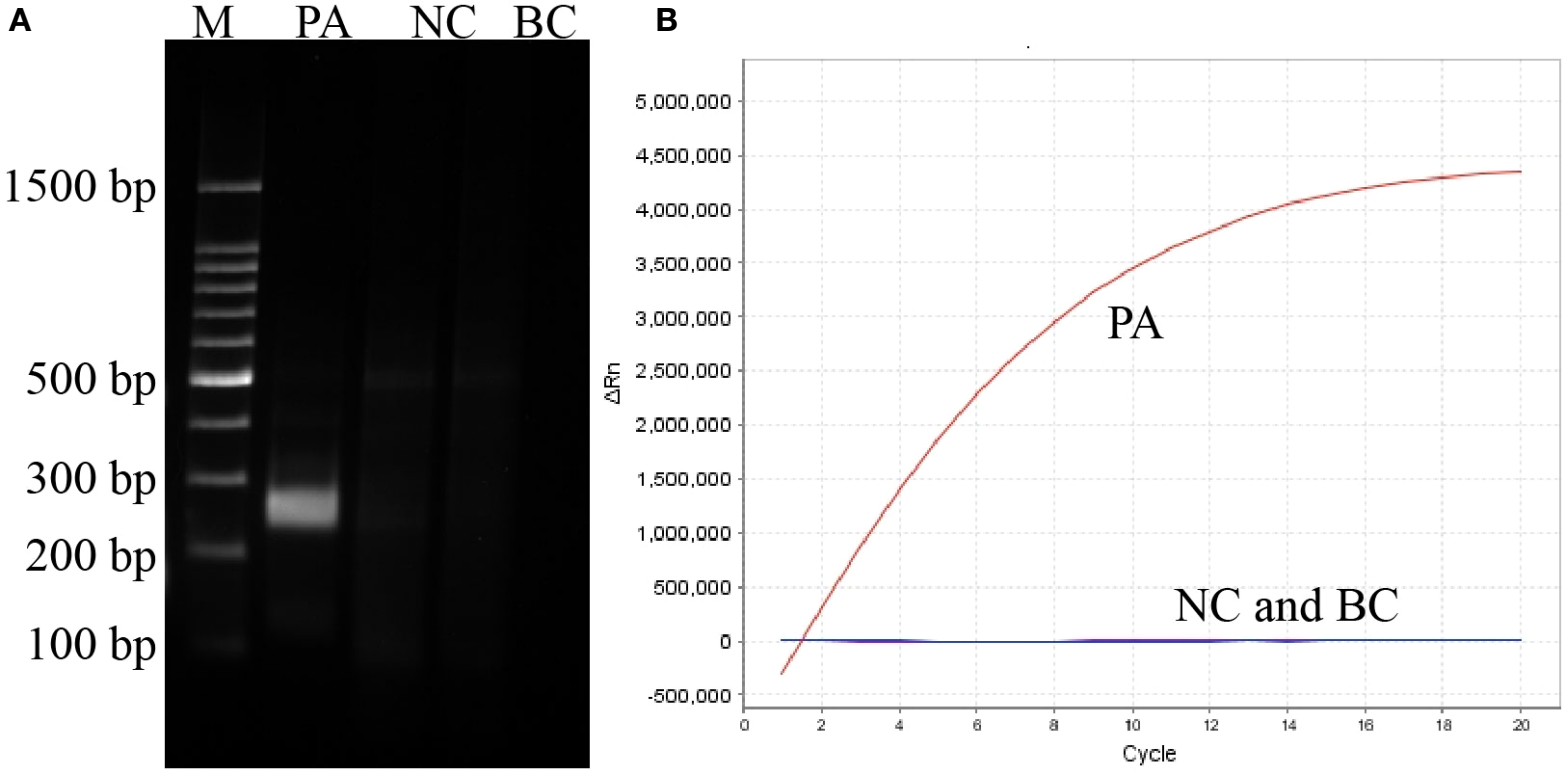
Establishment and confirmation of *P. aeruginosa*–CRISPR–RPA for *P. aeruginosa* detection. **(A)** RPA products amplified with primer pair F2/R6 are detected by agarose gel electrophoresis. **(B)** CRISPR-Cas12a biosensing system is used for detection of the target product. PC, positive control of *P. aeruginosa* strain; NC, negative control of *Klebsiella pneumonia*; BC, blank control of distilled water.

With primer pair F2/R6, only the reaction tube with *P. aeruginosa* genomic DNA as template displayed a bright target band in the gel electrophoresis image and generated fluorescence, whereas no band or fluorescence was produced in the negative control (with genomic DNA of *Escherichia coli* as template) and blank control (DW) reaction products (**Figure 2**). Thus, the primer F2/R6 and the developed *P. aeruginosa*–CRISPR–RPA assay were available to detect *P. aeruginosa* strains.

### Optimization conditions for *P. aeruginosa*–CRISPR–RPA assay

The *P. aeruginosa*–CRISPR–RPA assay was a two-step method and consisted of RPA pre-amplification and CRISPR-Cas12a detection two procedures. First, the optimum temperature and time of RPA pre-amplification step were determined by performing RPA reaction at temperatures ranging from 37°C to 42°C (with 1°C interval) and with time from 10 to 40 min (with 10 min interval), respectively. As shown in **Figures S2A, B**, a reaction temperature of 42°C and a reaction time of 20 min exhibited better amplification efficiency and thus were more suitable for RPA reaction. Then, the CRISPR-Cas12a detection step was optimized by performing tans-cleavage reaction within 50- or 100-µL volume, at 37 to 42°C (with 1°C interval) and with time from 10 to 20 min (with 5 min interval), respectively, and the trans-cleavage efficiency under different conditions was compared. According to the fluorescence intensity images (**Figures S2C–E**), a reaction volume of 100 µL, a temperature of 37°C, and a reaction time of 10 min generated higher fluorescence intensity and thus were better candidates for CRISPR-Cas12a detection for *P. aeruginosa*–RPA products. Therefore, 42°C and 30 min for *P. aeruginosa*–RPA reaction as well as 100 µL of reaction mixture, 37°C, and 10 min for CRISPR-Cas12a detection of *P. aeruginosa*–RPA products were selected for the subsequent *P. aeruginosa*–CRISPR–RPA assay. Moreover, the functional components within the reaction mixture were also confirmed by detecting the fluorescence intensity with each combination. As shown in **Figure S3**, only the combination contained all the components (CRISPR-Cas12a, crRNA, probe, and RPA product) displayed a positive result.

### Sensitivity and specificity evaluation of the *P. aeruginosa*–CRISPR–RPA assay

**Figure 3:**
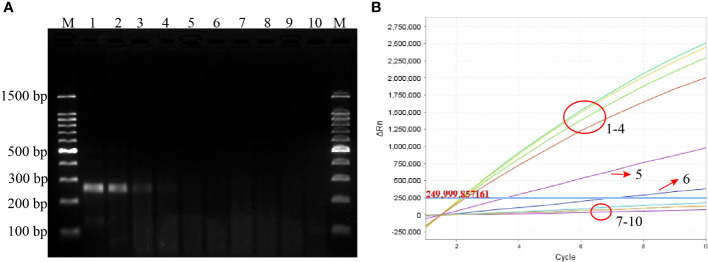
Sensitivity evaluation of the *P. aeruginosa*–CRISPR–RPA assay. Sensitivity assay was performed by using agarose gel electrophoresis **(A)** and CRISPR-Cas12a biosensing system **(B)** to detect the RPA products using gradient-diluted *P. aeruginosa* genomic DNA. Numbers 1–8 refer to the serial dilutions of *P. aeruginosa* genomic DNA from 6 ng to 0.6 fg, number 9 refers to the negative control (*E. coli*), and number 10 refers to the blank control (DW). Fluorescence intensity higher than 250,000 was considered as positive result.

The sensitivity of the *P. aeruginosa*–CRISPR–RPA assay was estimated by detecting the serially diluted genomic DNA of *P. aeruginosa* strain. As shown in **Figure 3B**, when dilution concentration exceeded 60 fg, apparent fluorescence intensity was generated by the real-time fluorescence detector, indicating that the *P. aeruginosa*–CRISPR–RPA assay was able to detect low as 60 fg *P. aeruginosa* genomic DNA per reaction. Compared with agarose gel electrophoresis after *P. aeruginosa*–RPA pre-amplification (6 pg, **Figure 3A**), the *P. aeruginosa*–CRISPR–RPA assay was obviously more sensitive to diagnose *P. aeruginosa* infection.

**Figure 4:**
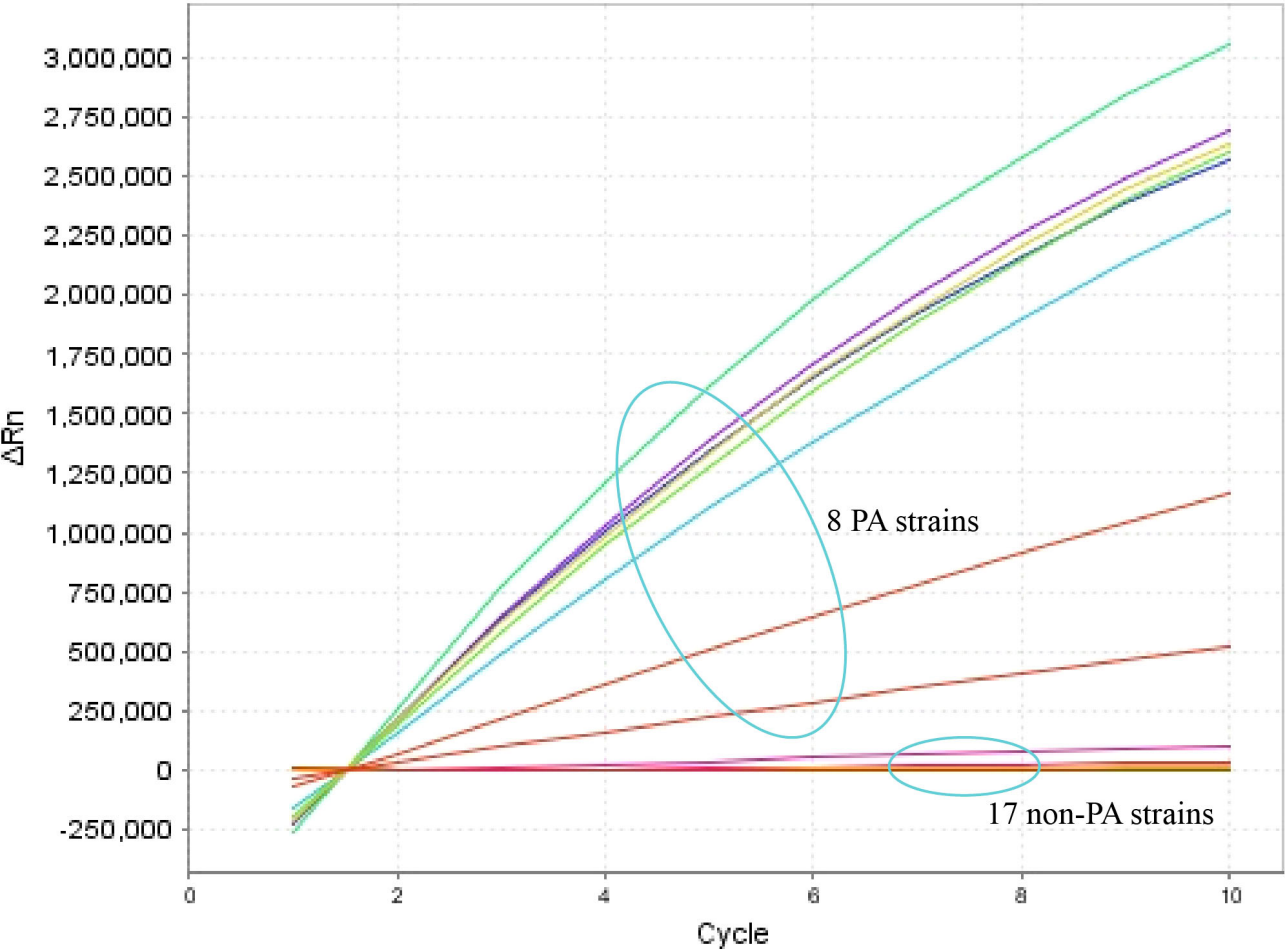
Specificity evaluation of the *P. aeruginosa*–CRISPR–RPA assay. Specificity assay was conducted by detecting the fluorescence intensity of 17 non–*P. aeruginosa* strains and eight *P. aeruginosa* strains by the real-time fluorescence detector. Fluorescence intensity higher than 250,000 was considered as positive result.

The specificity of the *P. aeruginosa*–CRISPR–RPA assay was assessed by using genomic DNA templates extracted from 17 non*–P. aeruginosa* strains. The result of fluorescence detector indicated that no fluorescence was generated from the 17 non*–P. aeruginosa* strains and the blank control (DW), whereas the eight *P. aeruginosa* strains produced significant fluorescence (**Figure 4**). Thus, the *P. aeruginosa*–CRISPR–RPA assay did not cross-react with other common respiratory pathogens, indicating a high specificity (100%).

### Clinical validity of the *P. aeruginosa*–CRISPR–RPA assay

**Figure 5:**
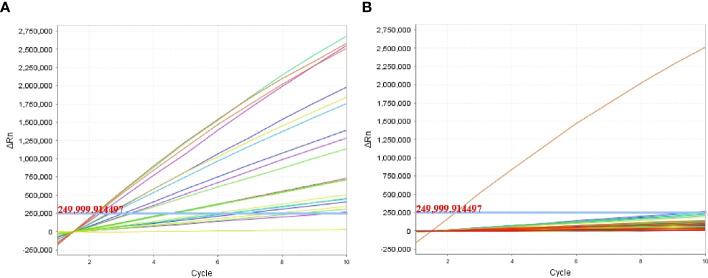
Clinical validity of the *P. aeruginosa*–CRISPR–RPA assay. A total of 96 BALF samples initial diagnosed by microfluidic chip (MFC) method were examined by the *P. aeruginosa*–CRISPR–RPA assay to confirm its application in clinical settings. Fluorescence intensity of the 19 P*. aeruginosa*–positive samples **(A)** and 77 P*. aeruginosa*–negative samples **(B)** were reported by the real-time fluorescence detector. Fluorescence intensity higher than 250,000 was considered as positive result.

To examine the performance of the *P. aeruginosa*–CRISPR–RPA assay in clinical practice, the detection platform was applied in clinical samples from patients with suspected respiratory infection. Of the 96 BALF samples, 19 were diagnosed as *P. aeruginosa*–positive, which were detected as *P. aeruginosa*–positive by the MFC method as well; whereas, the other 77 samples were negative for *P. aeruginosa* by both the *P. aeruginosa*–CRISPR–RPA assay and MFC method (**Figure 5**; **Table 3**). The detection result of the 96 clinical samples was identical between the *P. aeruginosa*–CRISPR–RPA assay and MFC method. These results demonstrated that the *P. aeruginosa*–CRISPR–RPA assay developed here was a reliable tool for *P. aeruginosa* detection in clinical settings.

**Table 3:** Performance comparison between the *P. aeruginosa*–CRISPR–RPA assay and microfluidic chip (MFC) method for *P. aeruginosa* detection in clinical samples.

Methods	*P. aeruginosa*–CRISPR–RPA		Comparison of two methods		
MFC	Positive	Negative	Sensitivity (%)	Specificity (%)	Kappa
Positive	19	0	100	100	1
Negative	0	77			

## Discussion

*P. aeruginosa* was one of the most common pathogens of hospital-acquired pneumonia (HAP) (16.9%–22.0%) (Moradali et al., 2017; Reynolds and Kollef, 2021) and also accounted for at least 1.0% of community-acquired pneumonia (Fine et al., 1996). Moreover, it was reported that 27.7% of the *P. aeruginosa* strains isolated from patient with HAP admitted in Intensive Care Unit (ICU) were of carbapenem resistance (Botelho et al., 2019). The high disease burden caused by *P. aeruginosa* and the increasing trend of antimicrobial resistance even multi-drug resistance of *P. aeruginosa* strains challenged the public health globally, and improvements to increase *P. aeruginosa* identification rate and time were urgently needed.

The CRISPR-Cas biosensing system has inspired numerous research activity in the diagnostic area on nucleic acid detection platform development (Li et al., 2019; Li et al., 2021; Jia et al., 2023), and the recently well-developed nucleic acid detection methods (such as SHERLOCK, HOLMES, DETECTR, and HUDSON) have manifested this. These methods were mainly developed with various CRISPR-Cas effectors (such as 12a, 12b, 13a, and 13b), which normally possess trans-cleavage activity, and activation of the trans-cleavage activity commonly required the formation of Cas effector/crRNA/target DNA ternary complex (Li et al., 2018a; Li et al., 2019). For example, the CRISPR-Cas12a effector could target DNA and trans-cleave any collateral ssDNA (Zetsche et al., 2015). Only after recognizing the target sequence that complementary to the crRNA sequence and juxtaposed with a suitable protospacer-adjacent motif (PAM, TTTN), the trans-cleavage activity of the CRISPR-Cas12a effector was able to be activated, following the paired fluorescence/quencher-labeled ssDNA probe cleaved and a fluorescent readout generated, which could be monitored by the real-time fluorescence detector (Li et al., 2018b). Owing to its merits of being highly efficient, sensitive, ultra-specific, and time-efficient, the CRISPR-Cas biosensing system has attracted much attention for its application in molecular diagnostic field. Therefore, in this study, we integrated the CRISPR-Cas12a biosensing system with RPA isothermal amplification techniques to optimize the *P. aeruginosa* identification rate and efficiency.

Compared with other nucleic acid amplification techniques, RPA was more preferable due to its simplicity, sensitivity, extremely rapidity, operation at low and constant temperature and with simple instruments, and no need for multiple primers (Lobato and O'Sullivan, 2018). In this study, the RPA pre-amplification step could be completed within 20 min at 42°C only with a simple water bath that could sustain a constant temperature. Because the reagents of RPA were freeze-dried and stored in the reaction tube, the RPA kit was especially convenient to store and employed. Thus, in this study, by combining with CRISPR-Cas12a detection platform, the *P. aeruginosa*–CRISPR–RPA assay was able to be performed independent of sophisticated equipment and foregoing the need for maintenance of a cold chain, which were attractive for use in point-of-care diagnostics and rural areas. Moreover, after optimization, the detection procedure of *P. aeruginosa*–CRISPR–RPA assay could be completed within half an hour, including 20 min for RPA reaction and 10 min for CRISPR-Cas12a detection, which was apparently rapid than that of PCR-based method and other isothermal amplification methods. In general, the *P. aeruginosa*–CRISPR–RPA assay in this study ensured its high sensitivity by pre-amplification the target nucleic acid using the attractive RPA method and guaranteed its high specificity with both the specific RPA primers and gRNA, together with paired fluorescence/quencher-labeled ssDNA probe, producing accurate and easy-to-interpret readouts.

The new established *P. aeruginosa*–CRISPR–RPA assay was proven sensitive to detect *P. aeruginosa* strains. It can detect as low as 60 fg (~8 copies) of *P. aeruginosa* genomic DNA per reaction, obviously more sensitive than the RPA-only method that detected by agarose gel electrophoresis method (6 pg). When compared with the previously reported *P. aeruginosa*–MCDA assay (100 fg) (Wang et al., 2020), the *P. aeruginosa*–CRISPR–RPA assay also exhibited higher sensitivity. However, the sensitivity of the *P. aeruginosa*–CRISPR–RPA assay was slight lower than the RPA-LFS assay developed by Yang et al. in 2021 (3.05 copies per reaction) (Yang et al., 2021). Thus, further optimization of the *P. aeruginosa*–CRISPR–RPA assay is still needed to improve the detection sensitivity, and more comparisons will be carried out to provide better reference to the clinicians for rapid and accurate diagnosis of *P. aeruginosa*–associated infections.

The specificity of the *P. aeruginosa*–CRISPR–RPA assay was evaluated by 17 non–*P. aeruginosa* strains, most of which were common respiratory pathogens. After detected by the *P. aeruginosa*–CRISPR–RPA assay, none of the 17 non–*P. aeruginosa* strains displayed a positive result except for the eight *P. aeruginosa* strains, manifesting that the *P. aeruginosa*–CRISPR–RPA assay was specific enough for diagnosis of *P. aeruginosa* infection. However, an obvious drawback of the specificity evaluation test was that no other members of genus *Pseudomonas* strains was tested here; thus, it will be supplemented if available in the future. After all, the *P. aeruginosa*–CRISPR–RPA assay was highly specific to detect *P. aeruginosa* strains and had no cross-reactivity with other pathogens.

Finally, the clinical validity of the *P. aeruginosa*–CRISPR–RPA assay was evaluated using 96 BALF samples initially diagnosed by MFC method. The *P. aeruginosa*–CRISPR–RPA assay reported 19 P*. aeruginosa*–positive samples and 77 negative samples, all of which was consisted with results by the MFC method, implying the *P. aeruginosa*–CRISPR–RPA assay was reliable for *P. aeruginosa* detection. Moreover, the *P. aeruginosa*–CRISPR–RPA assay was able to report the results of these clinical samples within half an hour, whereas that by MFC method needs about an hour, further demonstrating the superiority of the *P. aeruginosa*–CRISPR–RPA assay. It was well-known that conventional culture–based technique was more proper to be employed to validate the new established *P. aeruginosa*–CRISPR–RPA assay; however, no original clinical BALF samples was available; thus, comparison of the performance of this new method with culture-based technique could only be carried out in the future studies. Together, it could be concluded that the validity was a promising tool for the rapid and accurate diagnosis of *P. aeruginosa* infection.

Certainly, there are still some limitations in this study: (1) the genetic information of the eight *P. aeruginosa* strains was not available, which may affect the evaluation of the new established detection system in correctly *P. aeruginosa* identification; (2) the background fluorescence signal is occasional high, which may lead to false-positive results; (3) the carryover contamination cause by opening the RPA amplification tube may produce background signals; and (4) more clinical samples should be tested for clinical validation. Of course, there are also some improvements that can be made in the future, including using more genetically diverse strains for the method establishment and verification, interpreting the result of *P. aeruginosa*–CRISPR–RPA assay under blue light if the drawback of high background signals solved; moreover, conducting the whole detection procedure within one step if further optimizations were provided, which also can avoid the production of aerosol pollution.

In summary, we reported the development and validation of a CRISPR-Cas12a–based detection platform for *P. aeruginosa* identification and termed it *P. aeruginosa*–CRISPR–RPA assay. The two-step *P. aeruginosa*–CRISPR-RPA assay was capable of detecting *P. aeruginosa* only within half an hour with simple instruments. After detecting the serial dilutions of *P. aeruginosa* genomic DNA, other non–*P. aeruginosa* strains and clinical samples with the *P. aeruginosa*–CRISPR-RPA assay, it can be concluded that the *P. aeruginosa*–CRISPR–RPA assay possesses the merits of rapidity, reliability, easy to perform, higher sensitivity, and specificity. Thus, the *P. aeruginosa*–CRISPR–RPA assay established here was a reliable and promising tool for early and rapid diagnosis of *P. aeruginosa* infection and stop of its wide spread especially in the hospital settings.

## Data available statement

The original contributions presented in the study are included in the article/**Supplementary Material**. Further inquiries can be directed to the corresponding authors.

## Ethics statement

The studies involving humans were approved by ethic committee of Capital Institute of Pediatrics. The studies were conducted in accordance with the local legislation and institutional requirements. The human samples used in this study were acquired from primarily isolated as part of your previous study for which ethical approval was obtained. Written informed consent for participation was not required from the participants or the participants’ legal guardians/next of kin in accordance with the national legislation and institutional requirements.

## Author contributions

SL performed the experiments, analyzed the data, and drafted the manuscript. SH, FL, and YS helped perform the experiments and organize the data. JF, FX, NJ, XH, and CS provided experimental reagents and materials. JZ and YW supervised this study and revised the manuscript. DQ conceived, supervised and funded this study, and revised manuscript. All authors contributed to the article and approved the submitted version.
