# Supplementary material for: Rapid detection of Pseudomonas aeruginosa by recombinase polymerase amplification combined with CRISPR-Cas12a biosensing system
Source: Front Cell Infect Microbiol. 2023 Aug 10;13:1239269. doi: 10.3389/fcimb.2023.1239269 (PMC10449609; doi:10.3389/fcimb.2023.1239269)
Supplement: Supplementary file 1 [file DataSheet_1.docx]

**Title page**

**Rapid detection of *Pseudomonas aeruginosa* by recombinase polymerase amplification combined with CRISPR-Cas12a biosensing system**

Shuang Liu^1^, Siyuan Huang^1^, Fang Li^1^, Yuanyuan Sun^1^, Jin Fu^2^, Fei Xiao^2^, Nan Jia^2^, Xiaolan Huang^2^, Chunrong Sun^2^, Juan Zhou^2*^, Yi Wang^2*^, Dong Qu^1*^

^1^Department of Critical Medicine, Children’s Hospital Affiliated Capital Institute of Pediatrics, Beijing 100020, P.R. China.

^2^Experimental research center, Capital Institute of pediatrics, Beijing, 100020, P.R. China.

***Correspondence:**

**Dong Qu**, qudong2012@126.com (Handing the Correspondence)

**Yi Wang**, [wildwolf0101@163.com](mailto:wildwolf0101@163.com)

**Juan Zhou,** zhoujuan2015@126.com

**Figures**


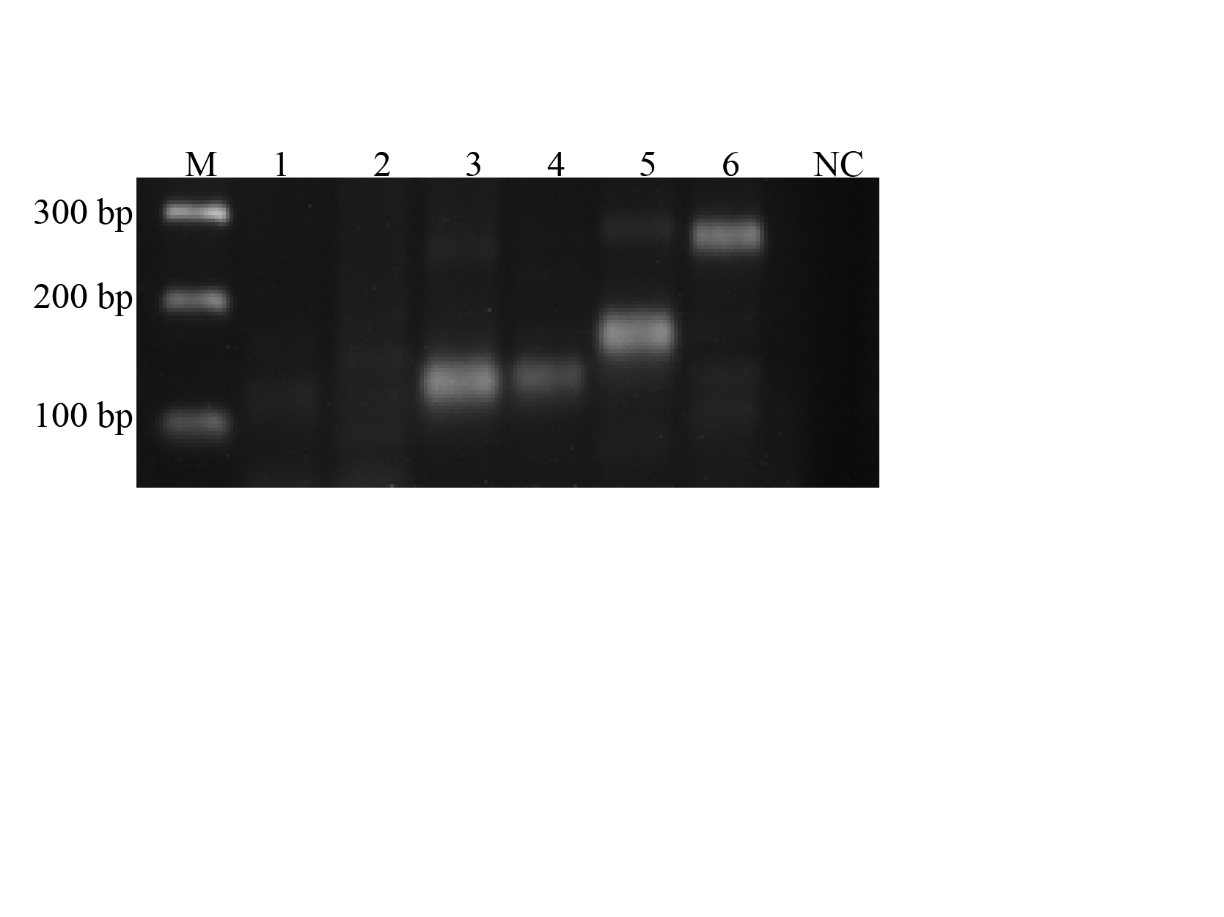
**Figure S1 Screen of RPA primers by agarose gel electrophoresis**. The RPA reaction was performed with 6 sets of primers. Set of primers: 1, F1/R1; 2, F1/R2; 3, F2/R3; 4, F2/R4; 5, F2/R5; 6, F2 and R6. NC, negative control.

**Figure S**
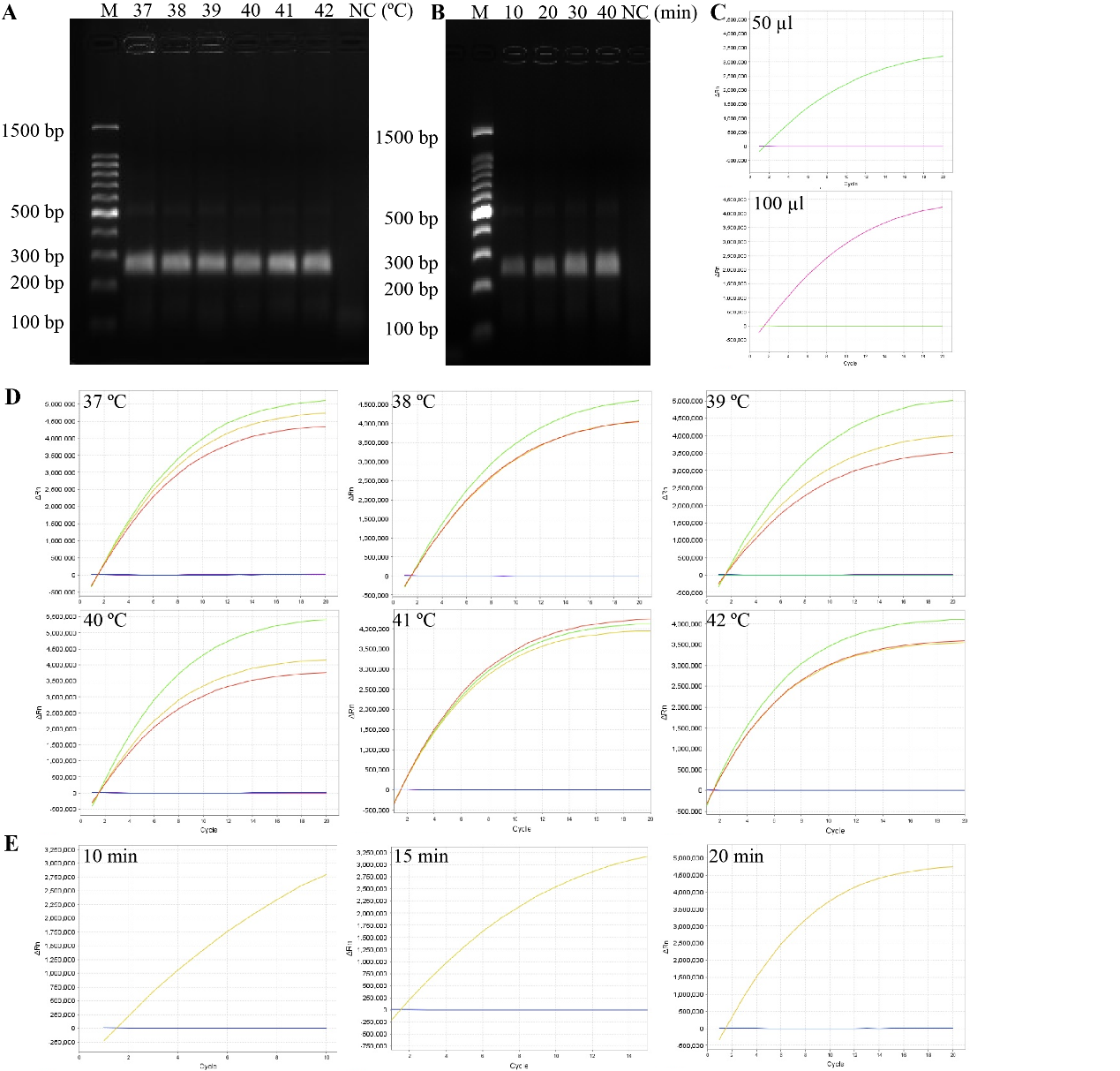
**2 Optimization of the *P. aeruginosa*-CRISPR-RPA assay.** Performance of RPA reaction at different temperatures from 37 to 42 °C (**A**) and different reaction time from 10 to 40 min (**B**) were tested by agarose gel electrophoresis. Performance of CRISPR-Cas12a detection reaction with different volume (50 μl versus 100 μl) (C), at different temperatures from 37 to 42 °C (**D**) and within different reaction time from 10 to 20 min (**E**) were detected by real-time fluorescence detector. NC, negative control.


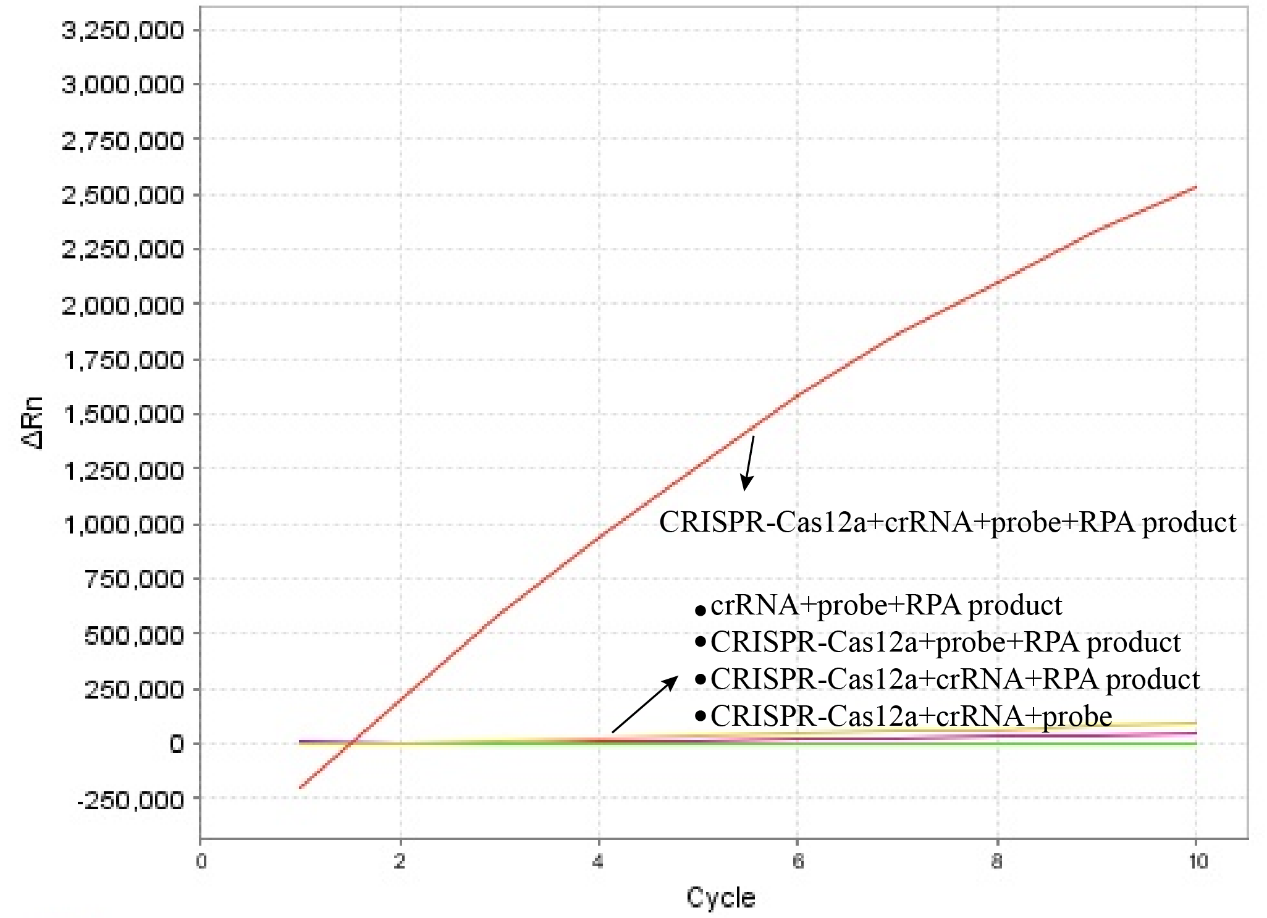
**Figure S3 Confirmation of functional components of the *P. aeruginosa*-CRISPR-RPA assay.** Different combinations of the components in the reaction mixture were detected by the real-time fluorescence detector.
